# Parotid pleomorphic adenoma: can a biopsy be omitted in the setting of a characteristic MRI?

**DOI:** 10.1007/s11282-026-00942-6

**Published:** 2026-06-22

**Authors:** Amy S. Wong, Pramit M. Phal, Michelle Thong, Dean P. McKenzie, Benjamin J. Dixon

**Affiliations:** 1 Department of Otolaryngology Head and Neck Surgery, Epworth HealthCare, Melbourne, VIC Australia; 2 Faculty of Medicine, Dentistry and Health Sciences, Department of Surgery, University of Melbourne, Melbourne, VIC 3010 Australia; 3 Present Address: Monash Imaging, Monash Health, 246 Clayton Road, Clayton, Melbourne, VIC 3168 Australia; 4 Radiology Victoria, Sonic Healthcare Australia Radiology, Melbourne, VIC 3121 Australia; 5 Department of Radiology, University of Melbourne, Melbourne, VIC 3010 Australia; 6 Department of Radiology, Alfred Health, Melbourne, VIC 3004 Australia; 7 Office for Research, Epworth HealthCare, Melbourne, VIC 3121 Australia; 8 Department of Epidemiology and Preventive Medicine, Monash University, Melbourne, VIC 3004 Australia

**Keywords:** Parotid pleomorphic adenoma, MRI, Fine-needle aspiration cytology

## Abstract

**Objective:**

Pleomorphic adenoma (PA) is the most common benign parotid tumour. Magnetic resonance imaging (MRI) and fine-needle aspiration cytology (FNAC) are routinely used during preoperative work up. However, there is insufficient evidence to support the use of MRI alone to diagnose a PA. This paper aims to evaluate the diagnostic performance of MRI and FNAC in the diagnosis of PA and develop an MRI assessment tool to potentially reduce an invasive biopsy.

**Methods:**

A retrospective cohort study was conducted in 155 patients with histopathologically confirmed parotid tumours with preoperative MRI and/or FNAC between 2015 and 2020. Two experienced head and neck radiologists, both with over 20 years’ experience, blinded to final histopathology, evaluated 100 MRIs. Diagnostic values for detecting PA with MRI and FNAC were evaluated. An MRI assessment tool was developed based on clinical criteria and MRI characteristics typical for a PA.

**Results:**

The sensitivity, specificity, positive predictive value (PPV), negative predictive value, (NPV) and diagnostic accuracy for predicting PA were 93%, 97%, 96%, 94% and 95% (Area under ROC curve (AUC) 0.95) for FNAC and 95%, 90%, 86%, 96% and 92% (AUC 0.93) for MRI respectively. There was no statistically significant difference between MRI and FNAC for diagnosing PA with regards to sensitivity (*p* = 0.628), specificity (*p* = 0.162) or overall accuracy (*p* = 0.361).

**Conclusion:**

MRI is a noninvasive imaging technique that can predict PA with similar sensitivity, specificity and diagnostic accuracy compared with FNAC. The PA MRI assessment tool aims to assist in surgical management decisions, while potentially reducing the need for an invasive biopsy.

## Introduction

Salivary gland neoplasms (SGNs) are rare, comprising 3% of all head and neck tumours and 0.5% of all malignancies [1]. Approximately 80% of SGNs originate in the parotid gland, and of these, 80% are benign, with pleomorphic adenoma (PA) being the most common [2]. Mucoepidermoid carcinoma (MEC) is the commonest primary parotid malignancy, and in Australia, the most common malignancy in the parotid gland is metastatic cutaneous squamous cell carcinoma (cSCC) [3, 4]. Due to the possibility of malignant transformation, complete surgical excision is recommended for a PA [5]. Minimally invasive parotidectomy such as extracapsular dissection has been increasingly adopted globally due to similar recurrence rates and lower morbidity compared with superficial parotidectomy and total parotidectomy [6]. However, this approach should be avoided where malignancy is suspected. As such, accurate preoperative investigations are paramount.

Both preoperative magnetic resonance imaging (MRI) and fine-needle aspiration cytology (FNAC) are frequently used during preoperative workup of a parotid tumour. However, a systematic review by Liu demonstrated FNAC is either non-diagnostic or indeterminate in 5.3% of cases, making preoperative diagnosis and surgical planning challenging [7]. Furthermore, FNAC is invasive, with potential risks of local infection, facial nerve damage or tumour cell spread and has limited use in deep lobe and small-sized lesions [8]. Its low sensitivity (78%) and risk of sampling error due to the operator-dependent nature of the procedure suggests that FNAC is only useful in limited circumstances. Conversely, MRI is gaining increasing popularity due to its accurate and non-invasive nature [7, 9–11].

MRI provides detailed anatomic information, soft tissue characterisation and is the preferred imaging modality in the evaluation of a suspected SGN including those located in the deep lobe [3, 12, 13]. It is able to define tumour margins, local extent of disease, pattern of infiltration and in cases of malignancy, assess perineural spread, marrow involvement and intracranial extension of tumour [13–15]. There is insufficient evidence to support the use of MRI alone in the preoperative workup of a parotid tumour. However, there is emerging evidence that MRI can be used to predict malignancy and even diagnose some parotid tumours such as PA [1, 16–19]. The objective of this paper is to determine whether FNAC can be safely avoided in patients with a characteristic MRI for PA to reduce an invasive biopsy prior to surgery. We aim to evaluate the diagnostic performance of MRI and FNAC in the diagnosis of a PA and develop a PA MRI assessment tool based on typical MRI characteristics of a PA.

## Materials and methods

**Fig. 1:**
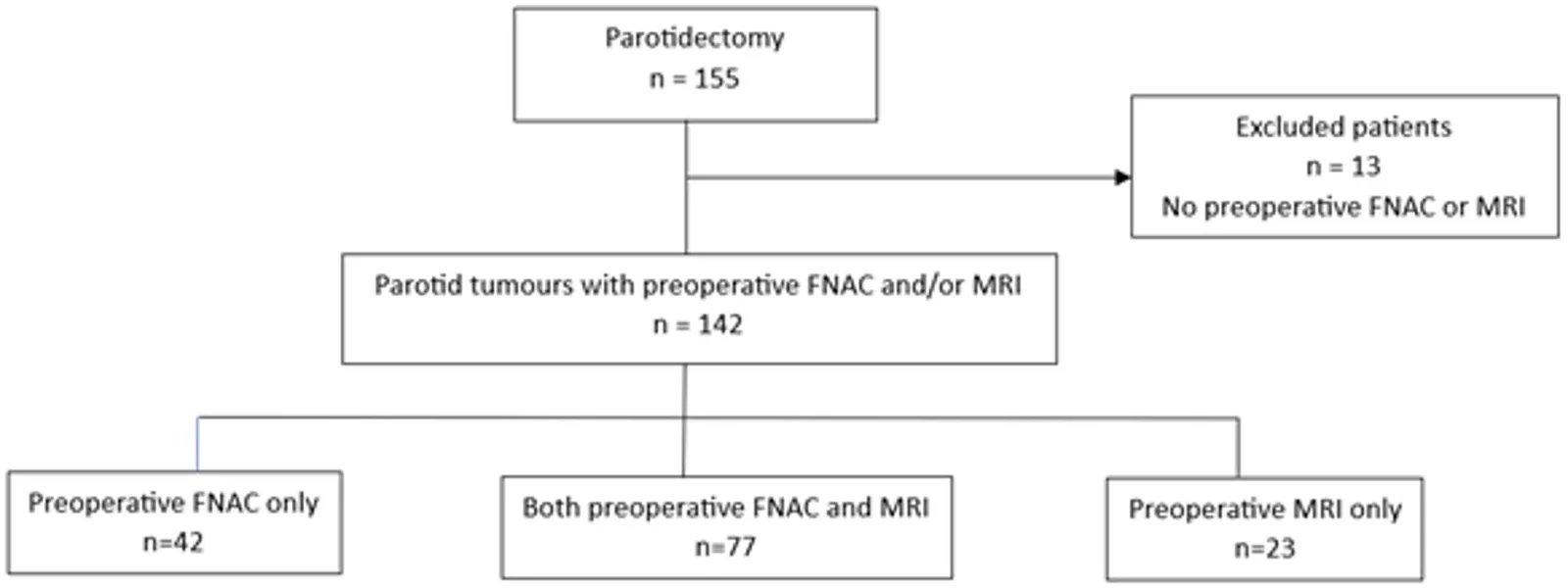
STARD flow chart

A retrospective cohort study was conducted in 155 consecutive patients who underwent parotidectomy between January 2015 and December 2020 at Epworth HealthCare, a not-for-profit organisation located in Melbourne. The project underwent governance review by the Office for Research at Epworth HealthCare (EH2019-474), who granted approval for the project. Informed consent was waived due to the retrospective nature of the study. The study was conducted according to the STARD guidelines (https://www.equator-network.org/reporting-guidelines/stard/) (Fig. 1). Both male and female patients of all age groups were retrospectively analysed.

*Inclusion criteria*

1. Patients who underwent either preoperative FNAC or MRI.
2. Appropriate MRI images available for evaluation.
3. FNAC reports available for review.

*Exclusion criteria*

1. Patients who did not have preoperative FNAC results or MRI images available for review.
2. Poor quality MRI images.

### MRI analysis

MRIs were evaluated independently by two head and neck radiologists, both with over 20 years’ experience, who were blinded to the final histopathology. 69 MRI images (69%) acquired at our institution were done using 3.0-T Skyra MRI unit (Siemens Medical Solutions, Erlangen, Germany) with a 20-channel receive only head and neck coil. MRI protocols consisted of turbo spine-echo (TSE) T1-weighted imaging (T1WI), contrast-enhanced T1WI with fat suppression, T2-weighted imaging (T2WI) with fat suppression and diffusion-weighted imaging (DWI). Details of specific MRI protocols are shown in the Appendix. 31 MRIs (31%) performed external to our organisation, on different imaging vendors at both 1.5 and 3.0T, were imported via picture archiving and communication system for review. DWI was used in 78 patients (78%). MRI was performed prior to FNAC in 33 out of 77 (42.9%) cases.

**Fig. 2:**
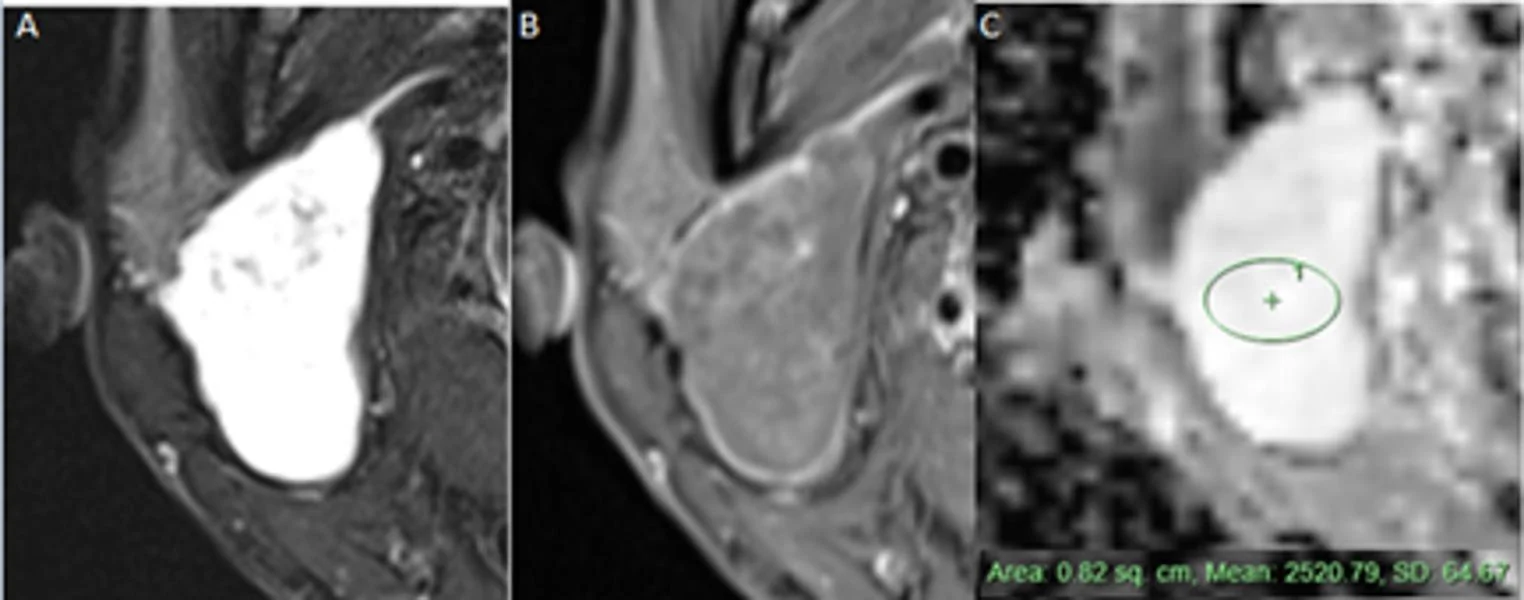
Mean apparent diffusion coefficient (ADC) value measurement (a: T2-weighted images (T2WI), b: contrast-enhanced T1-weighted images, c: ADC map). The ADC value was measured at 2.5 × 10^− 3^ mm^2^/s

MRI parameters reviewed include:

- Laterality (unilateral/bilateral).
- Tumour location (superficial/deep lobe/parotid tail/parapharyngeal).
- Shape (smooth/lobulated/irregular/spiculated).
- Size (≤ 2 cm/>2 and ≤4 cm/>4 cm).
- Margins (regular/irregular).
- Predominant T1 hyperintense / isointense /hypointense compared to parotid parenchyma.
- Predominant T2 hyperintense/isointense/hypointense compared to parotid parenchyma.
- T2 hypointense capsule (present/absent/partial).
- Contrast enhancement (homogeneous/heterogenous and mild/moderate/marked).
- Infiltration into adjacent structures (yes/no).
- Mean apparent diffusion coefficient (ADC) value within the solid component of the tumour measured using an elliptical region of interest (ROI) on ADC maps (Fig. 2).
- Presence of cystic areas within the tumour (yes/no).
- Presence of lymphadenopathy (yes/no).
- Presence of perineural spread (yes/no).

Any discrepancies between results were resolved via a consensus read to reach an inter-reader agreement.

### Fine-needle aspiration cytology

All fine-needle aspirations were performed under ultrasound guidance. Using aseptic technique, the mass was aspirated with two to three passes using a 21 to 25-gauge needle attached to a syringe. The aspirated cytological material was expelled onto glass slides and air-dried before being placed in a box for transportation to the Department of Pathology for review by a cytopathologist. The most probable preoperative cytological diagnosis was noted if available.

As the Milan System for Reporting Salivary Gland Cytopathology (MSRSGC) was introduced during the study period, where the Milan classification system was unavailable, cytopathology reports were reclassified. The six diagnostic categories consist of: *(I) non-diagnostic; (II) non-neoplastic; (III) atypia of undetermined significance (AUS); IVa) neoplasm: benign or IVb) neoplasm: SGN of uncertain malignant potential (SUMP); V) suspicious for malignancy; and VI) malignant.* MSRSGC V and VI were considered malignant while MSRSGC II and IVa were considered benign. MSRSGC I, III and IVb were considered indeterminate for the purpose of calculating diagnostic values.

### Histopathology

Histopathological diagnoses were examined by an experienced pathologist and served as the gold standard. Tumours were classified according to the 2022 World Health Organization (WHO) classification of salivary glands tumours (5th edition).

### Development of the PA MRI assessment tool

We developed a PA MRI assessment tool to identify a typical PA, primarily based upon clinical criteria, but also informed by the magnitude of odds ratios of different parameters from the analysis of our MRI results. The aim of the MRI assessment tool was to reduce an invasive procedure with biopsy compared with current standard of care. Hence, a conservative approach was used in the development of the algorithm. Misdiagnosis of malignancy as a PA was avoided to minimise the risk of malignant tumours undergoing inappropriate treatment with minimally invasive parotidectomy. Tumours with atypical characteristics of a PA, tumours with features suggestive of malignancy and indeterminate tumours were diverted back to the biopsy pathway. Validation of the assessment tool on a different dataset would be required to ensure clinical applicability.

### Statistical analysis

Descriptive analysis was used to summarise data. Continuous variables were presented as mean +/- standard deviation (SD) or median (interquartile range) based on the distribution of data. Categorical data were presented as numbers (percentages).

Diagnostic indices comprising sensitivity, specificity, positive predictive value (PPV), negative predictive value (NPV), overall diagnostic accuracy and area under the receiver operating characteristic (ROC) curve (AUC) were reported, along with 95% confidence intervals. Indeterminate results were considered negative as they failed to achieve the desired diagnosis. Diagnostic values were calculated with indeterminate results included to reflect more practical and clinically relevant findings [20, 21].

Statistical comparisons were made between the sensitivity, specificity and overall diagnostic accuracy values, employing logistic regression taking possible clustering within patients into account, as detailed in [22], due to some patients undergoing both MRI and FNAC.

Associations between potential individual MRI characteristics such as margins and malignancy and PA vs. non-PA tumours were examined using logistic regression. Statistical analyses were undertaken with and without adjusting for potential clustering for the two patients with two tumours. As the results were substantively the same, only the unadjusted analyses were reported. Inter-reader agreement Kappa (κ) and intraclass correlation coefficient (ICC) for independent MRI interpretations prior to consensus were analysed. ICC analysis employed a two-way mixed effects model, with patients representing a random effect, the latter calculated using a two-way model, with patients and raters treated as random effects and measuring absolute agreement. Statistical analysis was conducted using Stata 19 (Stata Corporation, College Station, Texas, 2025).

## Results

### Patient characteristics

There were 140 patients, 2 of whom had bilateral tumours, comprising 74 males (52.9%) and 66 females (47.1%). Mean age was 57 years (range, 16–92 years; SD, 17.8 years). The left and right parotid glands were equally involved (50.7% and 49.3% respectively).

### Tumour characteristics

Of the 142 parotidectomies that were performed, there was a total of 49 malignant (34.5%) and 93 benign tumours (65.5%). The most common tumour was PA (43.7%), followed by metastatic cSCC (19.7%). One patient had bilateral PA and one had bilateral Warthin’s tumour. Tumour characteristics and histopathological subtypes can be seen in Table 1.

**Table 1:** Tumour characteristics and histopathological subtypes

Histology		*N* = 142, n (%)
*Benign*		
Pleomorphic adenoma		62 (43.7%)
Warthin’s tumour		10 (7.0%)
Oncocytoma		8 (5.6%)
Basal cell adenoma		3 (2.1%)
Myoepithelioma		2 (1.4%)
Lymphoepithelial cyst		4 (2.8%)
Other		4 (2.8%)
*Malignant*		
Cutaneous squamous cell carcinoma		28 (19.7%)
Mucoepidermoid carcinoma		5 (3.5%)
Adenoid cystic carcinoma		3 (2.1%)
Mammary analogue secretory carcinoma		2 (1.4%)
Adenocarcinoma		2 (1.4%)
Carcinoma ex-pleomorphic adenoma		2 (1.4%)
Acinic cell carcinoma		1 (0.7%)
Basal cell carcinoma		1 (0.7%)
Salivary duct carcinoma		1 (0.7%)
Epithelial-myoepithelial carcinoma		1 (0.7%)
Mucosa-associated lymphoid tissue lymphoma		1 (0.7%)
Oncocytic carcinoma		1 (0.7%)
Merkel cell carcinoma		1 (0.7%)

### Preoperative FNAC and MRI

**Fig. 3:**
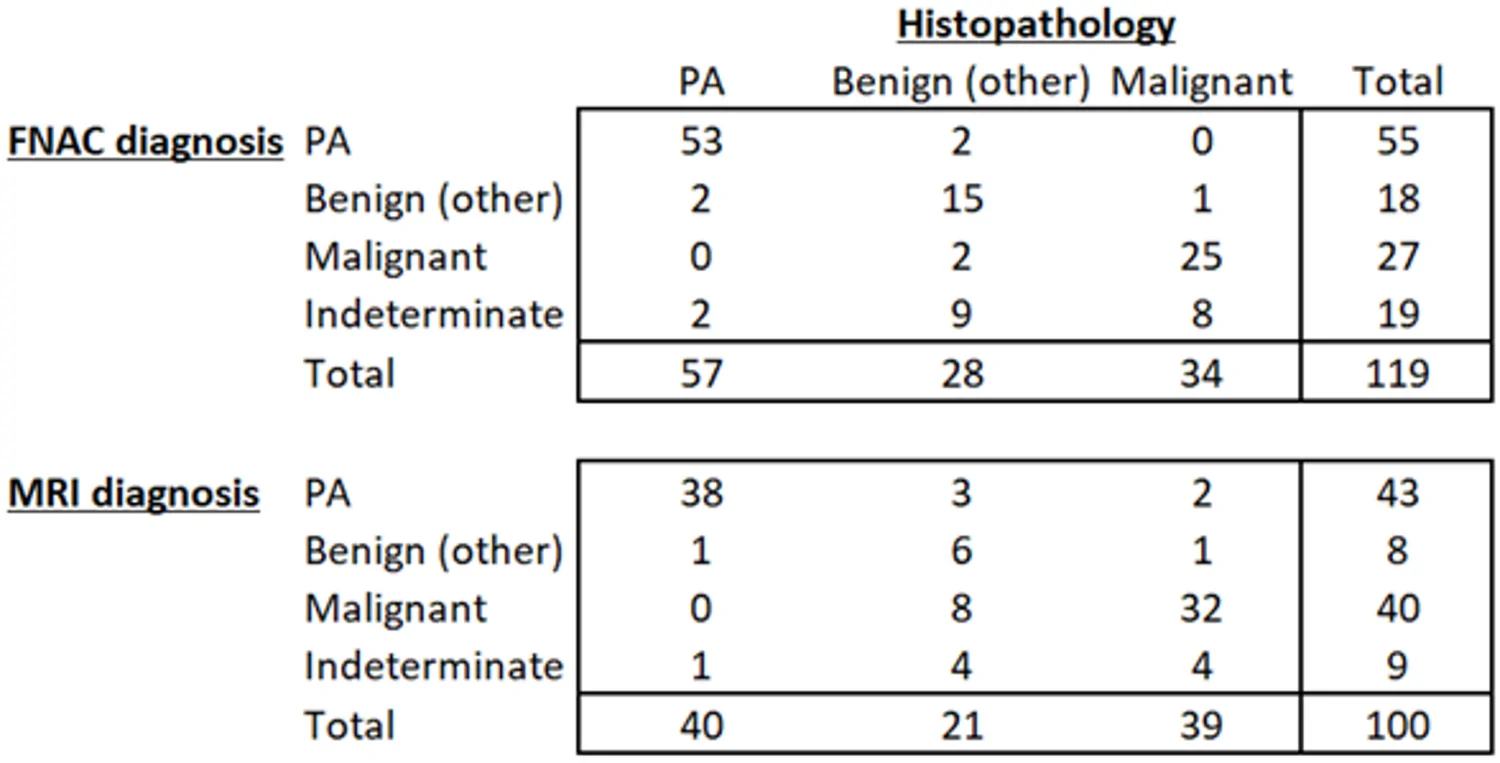
Distribution of FNAC and MRI results for pleomorphic adenoma and other benign and malignant tumours

119 parotid lesions underwent preoperative FNAC and 100 underwent preoperative MRI. Of these, 77 had both preoperative MRI and FNAC. The distribution of FNAC and MRI results for PA, other benign tumours and malignant tumours can be seen in Fig. 3. Indeterminate FNAC samples reached 16% overall. 6.7% were non-diagnostic (MSRSGC I) and 9.2% were indeterminate (MSRSGC III and IVb). Indeterminate cases on MRI reached 9% in our series.

### Diagnostic values

The diagnostic values for diagnosing PA and malignancy for the full cohort and those who underwent both FNAC and MRI are shown in Table 2.

**Table 2:** Diagnostic values of FNAC and MRI for a diagnosis of PA and malignancy for the full cohort and those who underwent both FNAC and MRI

Diagnostic value	Full cohort (95% CI)		Cases with both FNAC and MRI (95% CI)	
	FNAC (*n* = 119)	MRI (*n* = 100)	FNAC (*n* = 77)	MRI (*n* = 77)
*Pleomorphic adenoma*				
Sensitivity	93.0% (83.0-98.1)	95.0% (83.1–99.4)	94.3% (80.8–99.3)	94.3% (80.8–99.3)
Specificity	96.7% (88.7–99.6)	90.0% (79.5–96.2)	97.6% (87.4–99.9)	85.7% (71.5–94.6)
PPV	96.4% (87.5–99.6)	86.4% (72.6–94.8)	97.1% (82.6–99.6)	84.6% (69.5–94.1)
NPV	93.7% (84.5–98.8)	96.4% (87.7–99.6)	95.3% (84.2–98.8)	94.7% (82.3–99.4)
Diagnostic accuracy	94.9% (89.3–98.1)	92.0% (84.8–96.5)	96.1% (89.0-99.2)	84.4% (74.4–91.7)
AUC	0.95 (0.91–0.99)	0.93 (0.87–0.98)	0.96 (0.91-1.00)	0.9 (0.83–0.97)
*Malignancy*				
Sensitivity	73.5% (55.6–87.1)	82.1% (66.5–92.5)	79.2% (57.9–92.9)	70.8% (48.9–87.4)
Specificity	97.6% (91.8–99.7)	86.9% (75.8–94.2)	100.0% (93.3–100.0)	90.6% (79.3–96.9)
PPV	92.6% (75.7–99.1)	80.0% (64.4–90.9)	100.0% (82.4–100.0)	77.3% (54.6–92.2)
NPV	90.1% (82.1–95.4)	88.3% (77.4–95.2)	91.4% (82.3–95.9)	87.3% (75.5–94.7)
Diagnostic accuracy	90.8% (84.1–95.3)	85.0% (76.5–91.4)	93.5% (85.5–97.9)	84.4% (74.4–91.7)
AUC	0.86 (0.78–0.93)	0.85 (0.77–0.92)	0.90 (0.81–0.98)	0.81 (0.71–0.91)

PPV, positive predictive value; NPV, negative predictive value, AUC; area under the receiver-operating characteristics (ROC) curve

There was no statistically significant difference between MRI and FNAC in the total cohort for diagnosing PA on sensitivity (odds ratio OR = 1.43, 95% CI = 0.33–6.17, *p* = 0.628), specificity (OR = 0.3, 95% CI = 0.06–1.62, *p* = 0.162) or overall accuracy (OR = 0.61, 95% CI = 0.22–1.73, *p* = 0.363). With regards to malignancy, there was no statistically significant difference between the two tests on sensitivity (OR = 1.65, 95% CI = 0.50–5.38, *p* = 0.409) or overall accuracy (OR = 0.58, 95% CI = 0.25–1.32, *p* = 0.191). However, the specificity was higher for FNAC than for MRI, which was statistically significant (OR = 0.16, 95% CI = 0.03–0.78, *p* = 0.024). A subgroup analysis for the 77 cases with both MRI and FNAC demonstrated no significant difference in sensitivity (OR = 1.00, 95% CI = 0.23–444, *p* > 0.999), specificity (OR = 0.15, 95% CI = 0.02–1.38, *p* = 0.093) or diagnostic accuracy (OR = 0.35, 95% CI = 0.10–1.26, *p* = 0.107) for PA. Similarly, for malignancy, there was no difference between the two groups in sensitivity (OR = 0.64, 95% CI = 0.15–2.64, *p* = 0.536), specificity (OR = 0.15, 95% CI = 0-1.09, *p* = 0.063) or diagnostic accuracy (0.38, 95% CI 0.13–1.11, *p* = 0.078). Indeterminate results were included in analyses to reflect the recently revised Milan classification system to provide a more objective and practical measure for clinicians interpreting FNAC results [22].

### MRI characteristics

**Fig. 4:**
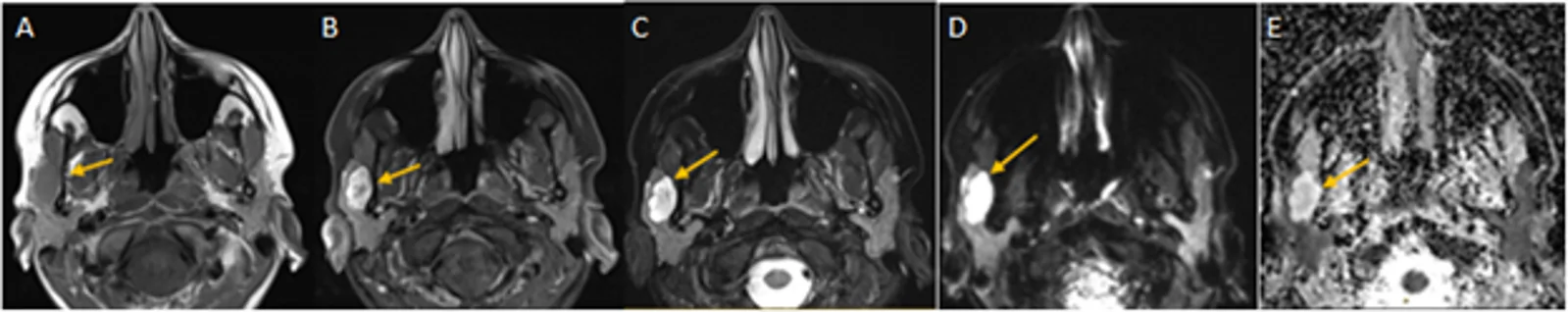
A-E. 26-year-old female with right pleomorphic adenoma, demonstrating typical imaging features. **A** Axial T1WI shows a well-defined lobulated tumour in the superficial lobe of the right parotid gland that is hypointense to the parotid gland (arrow). **B** Post contrast T1WI shows marked heterogenous enhancement (arrow). **C** T2WI shows predominant marked hyperintense signal intensity compared to the parotid gland and a well-encapsulated tumour (arrow). **D** DWI and **E** ADC maps show a lesion with high ADC values (arrows)

MRI parameters that were significantly associated with PA and benign tumours (*p* ≤ 0.05) include regular margins, smooth or lobulated shape, ADC ≥ 1.7 × 10^− 3^ mm^2^/s, T2 signal marked hyperintensity and an absence of perineural spread, lymphadenopathy and infiltrative growth (Fig. 4). In addition, compared with non-PA tumours (both benign and malignant), PA had T1 hypointensity, moderate to marked contrast enhancement and absent cystic areas that were statistically significant. On the other hand, malignancy was significantly associated with irregular margins, an irregular or spiculated shape and associated lymphadenopathy, perineural spread or infiltrative growth. Tables 3 and 4 show MRI variables and their odds ratios and statistical significance between benign and malignant tumours and between PA and non-PA tumours respectively. MRI parameters demonstrated a mean κ 0.67 (range 0.42–0.97; SD 0.17) while the ICC for size was 0.98 (95% CI = 0.97–0.99).

**Table 3:** Comparison of MRI findings between benign and malignant parotid tumours

MRI characteristic	Finding	Benign (*N* = 61)	Malignant (*N* = 39)	Odds ratio	95% CI	*p*-value
Infiltrative Growth	Present	4 (11.4)	31 (88.6)	**55.2**	15.4-198.1	**< 0.001**
	Absent	57 (87.7)	8 (12.3)			
T2 signal intensity	Hypointense/ Isointense/ hyperintense	29 (44.6)	36 (55.4)	**37.2**	4.8-289.7	**0.001**
	Marked hyperintense	30 (96.8)	1 (3.2)			
Tumour shape	Irregular / spiculated	2 (10.5)	17 (89.5)	**22.8**	4.9-106.8	**< 0.001**
	Smooth / lobulated	59 (72.8)	22 (27.2)			
Tumour margins	Irregular	6 (19.3)	25 (80.7)	**16.4**	5.63–47.6	**< 0.001**
	Regular	55 (79.7)	14 (20.3)			
ADC	< 1.7 × 10^− 3^ mm^2^/s	25 (49.0)	26 (51.0)	**13**	2.8–60.7	**0.001**
	≥ 1.7 × 10^− 3^ mm^2^/s	25 (92.6)	2 (7.4)			
Perineural spread	Present	0 (0)	5 (100)	12.3*	1.6-infinity	**0.013**
	Absent	61 (65.6)	32 (34.4)			
Lymphadenopathy	Present	4 (22.2)	14 (77.8)	**9.3**	2.8–31.6	**< 0.001**
	Absent	56 (72.7)	21 (27.3)			
Location	Superficial lobe	33 (50.0)	33 (50.0)	4.7	1.7–12.8	0.003
	Deep lobe involvement	28 (82.4)	6 (19.6)			
Cystic areas	Present	13 (50)	13 (50.0)	1.9	0.8–4.8	0.163
	Absent	46 (65.7)	24 (34.3)			
Contrast enhancement	Nil / minimal Moderate / Marked	26 (59.1) 35 (67.3)	18 (40.9) 17 (32.7)	1.4	0.6–3.3	0.405
Size	≤ 3 cm	52 (60.5)	34 (39.5)	1.2	0.4–3.8	0.786
	> 3 cm	9 (54.2)	5 (35.7)			
T1 signal intensity	Hypointense	55 (60.4)	36 (39.6)	1.1	0.3–4.9	0.909
	Isointense/Hyperintense	5 (62.5)	3 (37.5)			

*Exact logistic regression employed

P value statistically significant at ≤ 0.05

**Table 4:** Comparison of MRI findings between pleomorphic adenoma and other tumours (including benign and malignant tumours)

MRI characteristic	Finding	PA (*n* = 40)	Other tumours (*n* = 60)	Odds ratio	95% CI	*p*-value
Infiltrative Growth	Absent	39 (60.0)	26 (40.0)	**51.0**	6.6–396.0	**< 0.001**
	Present	1 (2.9)	34 (97.1)			
Tumour margins	Regular	39 (56.5)	30 (43.5)	**39.0**	5.0-302.5	**< 0.001**
	Irregular	1 (3.2)	30 (96.8)			
Lymphadenopathy	Absent	40 (52.0)	37 (48.0)	**26.4†**	4.3-infinity	**< 0.001**
	Present	0 (0)	18 (100)			
Tumour shape	Smooth/Lobulated	40 (49.4)	41 (50.6)	**25.2†**	4.1-infinity	**< 0.001**
	Spiculated/Irregular	0 (0)	19 (100)			
ADC	≥ 1.7 × 10^− 3^ mm^2^/s	23 (85.2)	4 (14.8)	**23.6**	6.6–83.7	**< 0.001**
	< 1.7 × 10^− 3^ mm^2^/s	10 (19.6)	41 (80.4)			
T2 signal intensity	Marked hyperintense	25 (80.7)	6 (19.3)	**13.9**	4.8–40.2	**< 0.001**
	Hypointense/ Isointense/ hyperintense	15 (23.1)	50 (76.9)			
T1 signal intensity	Hypointense	40 (44.0)	51 (56.0)	8.4*	1.2-infinity	**0.026**
	Isointense/Hyperintense	0 (0)	8 (100)			
Cystic areas	Absent	36 (51.4)	34 (48.6)	**5.8**	1.8–18.6	**0.003**
	Present	4 (15.4)	22 (84.6)			
Contrast enhancement	Moderate / Marked	29 (55.8)	23 (44.2)	**3.8**	1.6–9.1	**0.003**
	Nil / minimal	11 (25.0)	33 (75.0)			
Perineural spread	Absent	40 (43.0)	53 (57.0)	4.9*	0.6-infinity	0.135
	Present	0 (0)	5 (100)			
Location	Deep lobe involvement	17 (50.0)	17 (50.0)	1.9	0.8–4.3	0.145
	Superficial lobe	23 (34.8)	43 (65.2)			
Size	> 3 cm	6 (42.9)	8 (57.1)	1.2	0.4–3.6	0.814
	≤ 3 cm	34 (39.5)	52 (60.5)			

PA, pleomorphic adenoma

*Exact logistic regression employed

P value statistically significant at ≤ 0.05

All PA in our series were smooth or lobulated in shape with no lymphadenopathy. However, one PA had irregular margins and an infiltrative growth pattern, related to a biopsy performed prior to MRI. Compared with non-PA tumours, the majority of PA had a statistically significant high ADC value of ≥ 1.7 × 10^− 3^ mm^2^/s (85.2% vs. 14.8%, OR = 23.6, 95% CI 6.6–83.7, *p* < 0.001) and marked hyperintensity on T2WI (80.7 vs. 19.4%, OR 13.9, 95% CI 4.8–40.2, *P* < 0.001). Only non-PA tumours had tumours with significant T1 isointensity or hyperintensity (100% vs. 0%, OR 5.8, 95% CI 1.2-infinity, *p* = 0.026). Non-PA tumours also had a greater number of significant cystic areas (84.6% vs. 15.4%, OR 5.8, 95% CI 1.8–18.7, *p* = 0.003) and no or minimal contrast enhancement (75% vs. 25%, OR 3.8, 95% CI 1.6–9.1, *p* < 0.003).

### MRI assessment tool

**Fig. 5:**
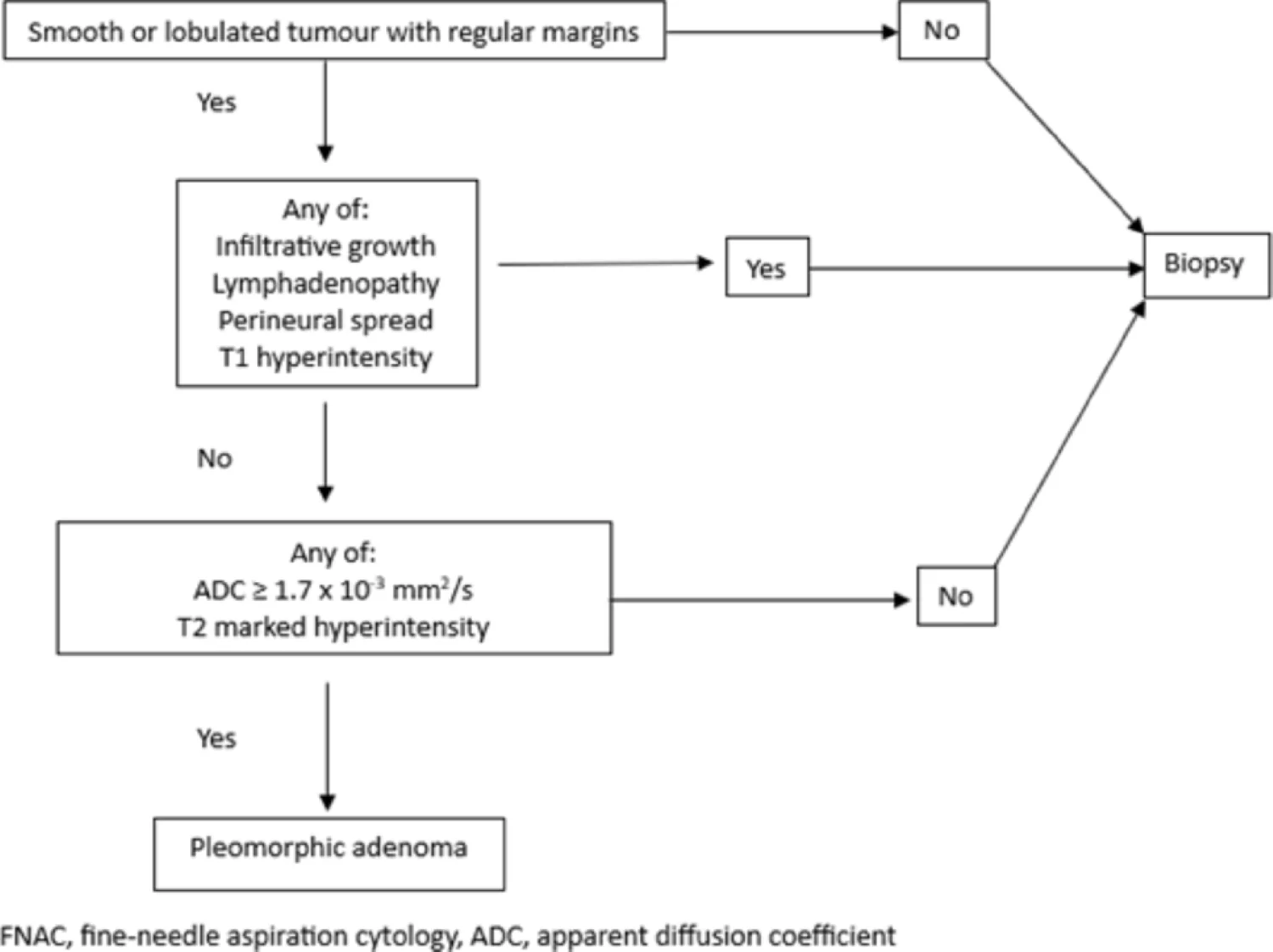
Proposed pleomorphic adenoma (PA) MRI assessment tool to identify a PA

We developed a PA MRI assessment tool (Fig. 5) to identify a typical PA, primarily based upon clinical criteria, informed by the magnitude of odds ratios of different parameters shown in Tables 3 and 4. Tumours with irregular margins which were not smooth or lobulated in shape would be referred for biopsy as these characteristics are atypical of a benign tumour. Tumours with MRI features suggestive of malignancy would also be diverted back to the biopsy pathway. After excluding these two groups of tumours, those with MRI features suggestive of PA (marked T2 hyperintensity and high mean ADC value ≥ 1.7 × 10^− 3^ mm^2^/s) received a final diagnosis of PA. MRIs with missing ADC values were treated as if the ADC criteria had not been met. Hence, for these tumours to receive a final diagnosis of a PA, the MRI must fulfil the final criteria of T2 marked hyperintensity, otherwise it would be diverted to the biopsy pathway.

**Fig. 6:**
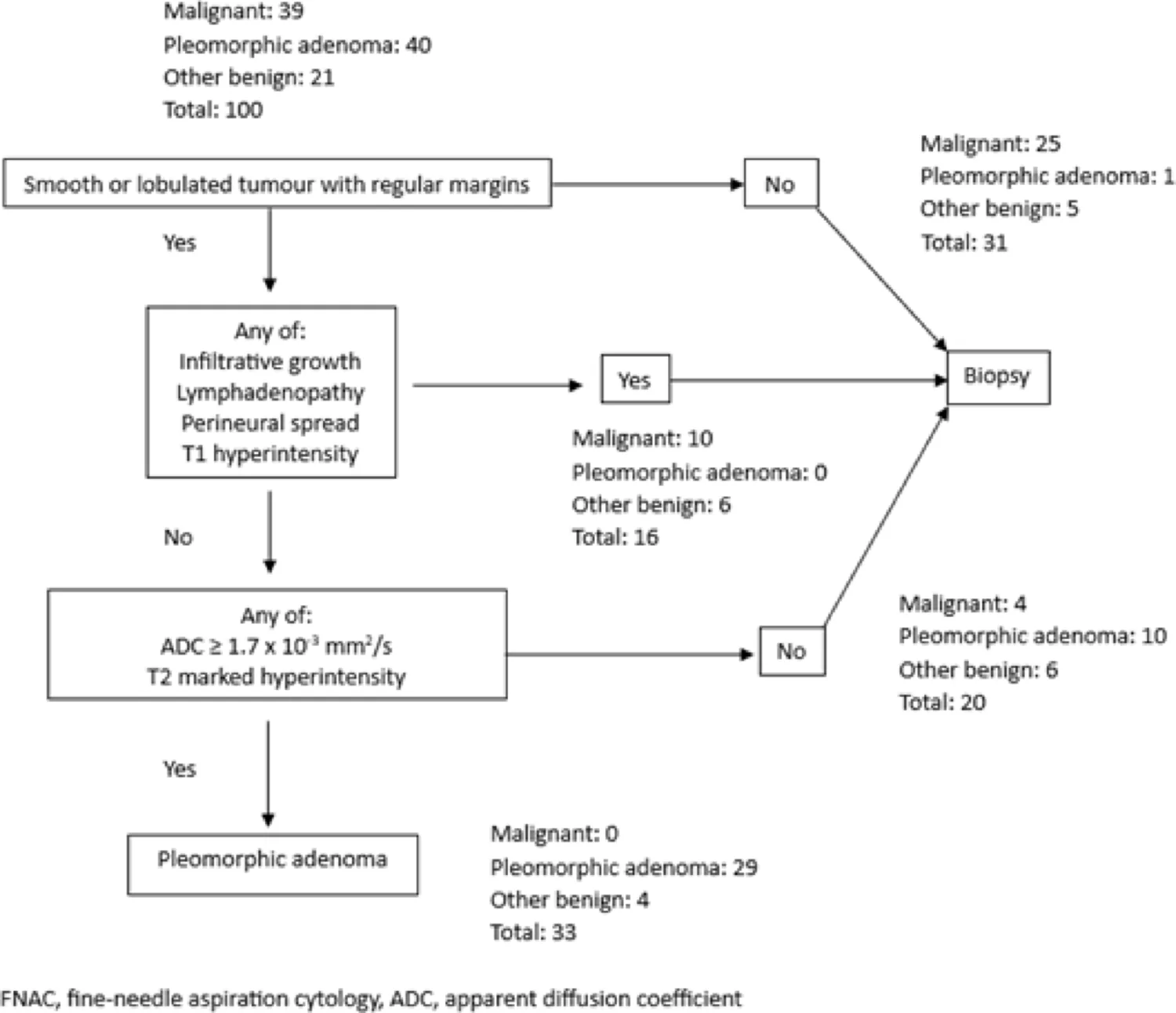
Retrospective results using the proposed pleomorphic adenoma (PA) MRI assessment tool with histopathological correlation

We retrospectively applied the above algorithm to our dataset to determine its performance. Results of the algorithm with histopathological results and diagnostic values along with 95% confidence intervals are shown in Fig. 6; Table 5 respectively.

**Table 5:** Diagnostic values of retrospectively applied MRI algorithm

Diagnostic value	MRI algorithm (CI)	MRI algorithm with missing ADC values excluded (CI)
Sensitivity	72.5% (56.1–85.4)	82.9% (66.4–93.4)
Specificity	93.3% (83.8–98.2)	93.2% (83.5–98.1)
PPV	87.9% (71.8–96.6)	87.9% (71.8–96.6)
NPV	83.6% (72.5–91.5)	90.2% (79.8–96.3)
Diagnostic accuracy	85.0% (76.5–91.4)	89.4% (81.3–94.8)
AUC	0.83 (0.75–0.91)	0.88 (0.81–0.95)

AUC, Area under the curve, CI, confidence interval

The algorithm diverted 3 groups of patients to the biopsy pathway: 31 cases without features of a benign tumour, 16 cases with features suggestive of malignancy and 20 cases without features suggestive of a PA. Of the 20 cases without features suggestive of a PA, 6 cases (5 PA, 1 benign tumour) had missing ADC values as DWI was not performed. The MRI assessment tool diagnosed 33 out of 100 tumours retrospectively as PA (33%), where a biopsy was not required. Of these, 29 were PA, and 4 were benign tumours. No malignancy was incorrectly diagnosed as a PA.

The sensitivity, specificity, PPV, NPV and diagnostic accuracy for diagnosing PA were 72.5%, 93.3%, 87.9%, 83.6% and 85% respectively. When the tumours with missing ADC values were excluded from the algorithm, diagnostic values yielded a sensitivity, specificity, PPV, NPV and diagnostic accuracy of 82.9%, 93.2%, 87.9%, 90.2% and 89.4% respectively.

## Discussion

PA is the most common benign tumour of the parotid gland that can be managed with minimally invasive parotid surgery. However, accurate preoperative diagnosis of PA is essential. Due to the invasive nature of FNAC, its low sensitivity rate (78%) and risk of sampling error due to the operator-dependent nature of the procedure, the potential to avoid an FNAC warrants consideration. MRI is an emerging noninvasive imaging technique that may be able to diagnose a PA, potentially deferring biopsy in selected cases pending prospective validation.

Our diagnostic values for diagnosing malignancy were consistent with the reported sensitivity, specificity and diagnostic accuracy of 40–90%, 81–100% and 84–97% respectively for FNAC in a meta-analysis by Liu; and the reported sensitivity, specificity, PPV, NPV and diagnostic accuracy of 73–87%, 77–100%, 76–100%, 95–97% and 93–96% respectively for conventional MRI in the literature [1, 16–19]. Few studies directly compared the diagnostic values of MRI and FNAC in the diagnosis of PA, with variable reporting of results [14, 16, 17, 19, 23–25]. Despite this, our results for diagnosing PA were in keeping with the reported sensitivity, specificity, PPV, NPV and diagnostic accuracy of 35–94%, 84–100%, 98%, 85–88% and 83–92% respectively for FNAC and the reported sensitivity, specificity and PPV of 44–94%, 84–100% and 88–96% respectively for MRI [3, 10, 18, 25–30].

Tryggvason demonstrated 100% sensitivity to diagnose PA with FNAC in 492 parotid tumours, but 6 out of 14 carcinoma ex-pleomorphic adenomas were incorrectly diagnosed as a PA, and a radiological diagnosis of PA was not performed in the imaging group [23]. Inohara demonstrated accurate cytological typing in 83% of FNAC samples for 40 PA but 8% were also incorrectly diagnosed as malignant and 10% of malignant tumours were incorrectly diagnosed as a PA [16]. They diagnosed tumours as benign or malignant on MRI, but a specific diagnosis of PA was not established. Paris excluded 4 indeterminate samples to identify 55 out of 60 (92%) PA with FNAC, and 39 out of 47 (83%) PA on MRI [14]. Yerli reported an accurate diagnosis of PA in 6 out of 6 cases with MRI and 4 out of 6 cases with FNAC [24]. Ikeda reported two findings that characterised PA on MRI: a low-signal capsule on T2WI which had a sensitivity 82%, specificity 85%, PPV 82% and NPV 84%; and a lobulated contour with a sensitivity 53%, specificity 84%, PPV 74% and NPV 67%, but an overall diagnosis of PA was not evaluated [27]. Laccourreye demonstrated a PPV of 88% for MRI and 85% for FNAC in the diagnosis of PA and found no difference in diagnostic performance with and without dynamic analysis on MRI which was not used in our study [25].

Nondiagnostic and indeterminate rates for FNAC in our study were consistent with the reported 5.3% and 14.7% respectively in the systematic review by Liu [7]. However, indeterminate rates for diagnosing PA with MRI have not been reported in published studies, hence our results could not be compared. Despite this, with indeterminate cases included, both MRI and FNAC performed well in our study. There was no statistically significant difference in the sensitivity, specificity or overall accuracy between the two modalities in diagnosing PA. Although our study suggests that MRI alone may be used for a diagnosis of a PA, statistical non-significance does not establish clinical equivalence, hence a larger sample size and a formal non-inferiority prospective randomised controlled study is required to confirm this hypothesis.

PAs are histologically characterised by epithelial, myoepithelial and stromal-myxoid components surrounded by a thin and incomplete fibrous capsule [31]. Imaging findings of PA are strongly influenced by cellular density, the proportion of epithelial and stromal components and the type of stromal components. Highly cellular tissues result in extracellular space narrowing, consequently exhibiting lower diffusion movement and thus have reduced ADC values. Typical PA has abundant myxochondroid stroma with scattered cellular components hence has extremely high ADC value with a reported range of 1.43 to 2.15 × 10^− 3^ mm^2^/s [2, 24, 32–34]. Conversely, predominant epithelial components with very scant or without stromal components (cellular variant PA) can cause intermediate ADC values, which resemble ADC values of intermediate-grade malignant SGN. Myoepitheliomas, formerly considered a variant of PA, may also be indistinguishable from PA using ADC [12]. Although necrosis and cystic changes are not specific features for differentiating benign and malignant tumours and are more commonly seen in Warthin’s tumour, cystic areas may also be seen in PA [35].

MRI features concerning for malignancy include irregular margins, T2WI hypointensity, low ADC values, infiltrative growth, perineural spread and lymphadenopathy [3, 12, 36]. Malignant tumours without cystic or necrotic components are more likely to have a lower mean ADC value, presumably owing to their more densely packed cellularity and higher nucleus to cytoplasm ratio [12]. A meta-analysis by Chen found DWI can rule out malignancy but is less able to confirm the presence of malignant disease [8]. Any tumours with concerning or atypical features of PA should be referred for a biopsy.

We developed an MRI assessment tool to diagnose a typical PA based on clinical grounds and MRI characteristics that were statistically significant for benign tumours and PA. When retrospectively applied to our dataset, 33 out of 100 cases (33%) were assessed as having typical MRI features of PA and a biopsy omitted. Of the 33 cases, 29 were accurately diagnosed out of 40 PA (72.5%). The remaining 4 cases consisted of 4 of the 21 benign tumours in the series (19%). Of the 11 PA (27.5%) which were diverted to a biopsy pathway, 5 had missing ADC values where DWI was not performed. With these cases excluded, a total of 6 cases out of 35 PA (17.5%) were diverted back to the biopsy pathway, which is the current standard of care. One had irregular margins and infiltrative growth due to a biopsy prior to MRI, which could have potentially been prevented by delaying FNAC until after the MRI. The remaining 5 PA were atypical, with ADC values ranging from 1.09 to 1.6 × 10^− 3^ mm^2^/s and T2 iso- or hyperintensity. Our assessment tool diagnosed 29 out of 40 patients with a PA (72.5%), saved 33 out of 100 patients (33%) from a biopsy and most importantly, had no misdiagnosis of malignancy as a PA. Although not a focus of our study, this has significant time, financial and economic impacts on the patient and the institution.

Our algorithm identified patients with a typical parotid PA who would be appropriate for minimally invasive parotid surgery, provided other surgeon-specific technical factors are also met, such as size, mobility and location within the parotid gland [6]. Although benign tumours may also be managed with limited surgery, due to overlapping MRI characteristics with some primary malignant tumours, a biopsy to confirm benignity in non-PA tumours is recommended. A watch-and-wait approach may also be suitable for those with non-malignant disease such as Warthin’s tumour. Conversely, all malignant tumours should be referred for a biopsy and limited parotid surgery avoided.

The aim of our algorithm was not to be the best performing diagnostic tool, but one that would be the safest for clinical use, to carefully select a group of PA that may not require a biopsy. We did this by designing a tool with high specificity to avoid inadvertently performing limited surgery on a malignant tumour. We undercalled indeterminate cases as PA and ensured they were excluded from limited surgery without a biopsy. Our assessment tool achieved our goal, demonstrating high specificity (93%) and PPV (88%), establishing its strength in confirming PA. As expected, sensitivity was lower at 73–83% with a NPV of 84–90%, as the algorithm undercalled atypical PA or indeterminate cases as a PA, to minimise false-positive rates. It should be noted, however, there is likely an overestimation of our reported diagnostic values because the assessment tool was evaluated on its own derivation cohort, which inherently introduces circularity and optimism bias. The assessment tool also underperformed compared to unstructured radiologist assessment (AUC 0.83–0.88 vs. 0.9–0.93) because of different metrics in its design, a cautious algorithm to only diagnose tumours which were certain to be a PA vs. diagnosing a parotid tumour with high diagnostic accuracy. The MRI tool is intended for use by clinicians and by both experienced and non-experienced radiologists to safely guide decision making. It can also help bridge the gap in centers lacking subspecialized head and neck neuroradiologists.

Few studies have developed an approach to the evaluation of a PA. Chakrabarty developed an MRI signature to diagnose PA which consisted of an encapsulated round-shaped tumour, well-defined lobulated margins, predominant T2 hyperintensity and heterogeneous enhancement or rim enhancement without any fat or T2 hypointense signal or diffusion restriction. Using these parameters, they demonstrated a sensitivity, specificity, PPV, NPV and accuracy of 82%, 96%, 69%, 98% and 94% respectively [11]. Our assessment tool performed better in PPV but not the other parameters. However, their sample size for PA was small, with only 13 PA out of 104 parotid tumours. Employing an automated variable selection procedure (backward stepwise regression), Abend found key predictors of PA included higher ADC values, T2 hyperintense signal, lobulated tumour contour and the absence of dumbbell shape in 86 out of 157 tumours [10]. Abend’s model achieved a sensitivity of 83% and specificity of 82%, with an area under the curve of 0.87. Our algorithm had higher specificity but not sensitivity than Abend’s model, likely due to the trade-off of differing priorities of not misdiagnosing a malignancy as a PA at the expense of missing a portion of PA. It should be noted however, our algorithm has not yet been applied on a different dataset, which limits the generalisability of these results. Further testing and external validation on a larger novel dataset using the Transparent Reporting of a multivariable prediction model for Individual Prognosis or Diagnosis (TRIPOD) is required [37].

Gokce, in a review, suggested an approach to MRI evaluation using T2WI hyperintensity, an intact hypointense rim on T2WI, ADC > 1.2 × 10^− 3^ mm^2^/s and T_peak_ > 120 s with no washout on dynamic contrast enhanced (DCE) MRI to suggest a PA [1]. However, DCE MRI is not routinely used in clinical practice, and the diagnostic performance of the approach was not reported. Zaghi developed a scoring algorithm yielding 95% specificity for the diagnosis of PA, which included bright signal on T2WI, sharp margins, heterogeneous nodular enhancement, lobulated contours and the presence of a dark rim on T2WI [18]. However, the algorithm achieved a low sensitivity of 44%, which would miss a large portion of PA.

### Study limitations

Our study has several limitations, including the retrospective nature of the study, a small sample size limited to patients treated at our institution, as well as a requirement for external validation. There are also several biases including selection, information, circularity and optimism bias. Our study was conducted in patients who underwent surgery at our institution, so the sample was biased towards patients with a neoplastic process, and particularly those with head and neck malignancy. Conditions where surgery was not the primary treatment such as hematological malignancy and inflammatory disease were underrepresented. Additionally, there was variability in MRI scanning equipment and protocols, as imaging data was collected from both in-house and externally using different scanners and acquisition parameters. Given ADC values can vary depending on technical factors, this heterogeneity may have influenced absolute ADC measurements. DWI was also not performed routinely by external vendors, which affected our results. However, this variability reflects real-world clinical practice and enhances the generalisability of our findings. Although previous multi-centre studies have demonstrated variation in ADC values across different platforms, relative trends demonstrated tend to persist.

MRI should ideally be performed prior to FNAC to avoid signal alterations which may hinder accurate MRI characterisation; however, this was not always possible [31]. In our study, 43% of MRIs were performed prior to FNAC, again reflecting real-world clinical practice. Studies have shown the role of advanced MRI techniques, such as DCE MRI, diffusion tensor imaging (DTI) and intravoxel incoherent motion (IVIM) for parotid tumour differentiation [3, 12, 31, 36]. However, these sequences are not routinely used at our institution for parotid imaging, hence were not incorporated into our algorithm. Finally, external validation of the MRI assessment tool on a novel dataset to assess whether our model can be reliably applied in clinical practice across different populations and settings is required, as is a prospective non-inferiority study.

## Conclusion

MRI is a non-invasive imaging technique that can predict parotid PA with similar sensitivity, specificity and diagnostic accuracy compared with FNAC, however prospective validation is required to determine whether MRI alone is sufficient for diagnosis of a PA. The PA MRI assessment tool developed in this study aims to safely guide management decisions, while potentially reducing the need for an invasive biopsy.

## Data Availability

Data is available on request from corresponding author AW.

## Appendix

**Table:**

Protocol	Matrix	FOV	Slice thickness/ gap	Slices	BW (kHz)	TR	TE	Time	NSA	Interpolation	Parallel imaging
T1 TSE axial	Deep Resolve Sharp & Boost 213 × 304	180 mm	4 mm / 0.4 mm	36	219	763	9.6	2.11	2	On 0.3 × 0.3 × 4	Grappa x 4
T1 C + TSE Dixon axial	Deep Resolve Sharp 202 × 256	180 mm	4 mm / 0.4 mm	36	354	685	10	3.04	1	On 0.31 × 0.31 × 4	Grappa x 2
T1 C + TSE Dixon coronal	Deep Resolve Sharp 202 × 256	180 mm	4 mm / 0.4 mm	32	305	960	11	3	1	On 0.31 × 0.31 × 4	Grappa x 2
T2 TSE Dixon axial	Deep Resolve Sharp 202 × 256	180 mm	4 mm / 0.4 mm	36	284	6490	66	2.44	1	On 0.28 × 0.28 × 4	Grappa x 2
T2 TSE Dixon coronal	Deep Resolve Sharp 202 × 256	180 mm	4 mm / 0.4 mm	32	320	6690	84	2.35	1	On 0.35 × 0.35 × 4	Grappa x 2
Resolve Diffusion axial	130 × 200 matrix	250 mm	4 mm / 0.4 mm	36	658	6540	64 / 107	3.49	b50_b800	None	Grappa x 2
*Options*											
T1 TSE sagittal	Deep Resolve Sharp & Boost 179 × 256	180 mm	4 mm / 0.4 mm	32	241	700	9.1	2.01	2	On 0.35 × 0.35 × 4	Grappa x 3
T1 C + TSE Dixon sagittal	Deep Resolve Sharp 179 × 256	180 mm	4 mm / 0.4 mm	32	305	771	11	3.02	1	On 0.35 × 0.35 × 4	Grappa x 2
T2 STIR SPACE Coronal	148 × 256	250	1.5 mm	88	349	3.24	3000	249	1	On 0.49 × 0.49 × 1.5	Grappa x 2

TSE, Turbo spin-echo; C+, contrast enhanced; STIR, Short Tau Inversion Recovery; FOV, Field of View; BW, Bandwidth; TR, Time to repetition; TE, Time to echo; NSA, Number of Signal Averaging

## Declarations

### Standards of reporting

The manuscript was prepared according to the STARD guidelines.

## References

[1] Gokce E. Multiparametric Magnetic Resonance Imaging for the Diagnosis and Differential Diagnosis of Parotid Gland Tumors. J Magn Reson Imaging; 2020. 10.1002/jmri.27061 32065489

[2] Kato H, et al. Current status of diffusion-weighted imaging in differentiating parotid tumors. Auris Nasus Larynx. 2023;50(2):187–95. 10.1016/j.anl.2022.07.002 35879151

[3] Stoia S, et al. Cross-sectional imaging and cytologic investigations in the preoperative diagnosis of parotid gland tumors - An updated literature review. Bosn J Basic Med Sci. 2021;21(1):19–32. 10.17305/bjbms.2020.5028 PMC7861630 32893758

[4] Iyer NG, et al. Outcomes following parotidectomy for metastatic squamous cell carcinoma with microscopic residual disease: implications for facial nerve preservation. Head Neck. 2009;31(1):21–7. 10.1002/hed.20912 18767180

[5] Liu HT, et al. Evaluation of the effectiveness of superficial parotidectomy and partial superficial parotidectomy for benign parotid tumours: a meta-analysis. J Otolaryngol Head Neck Surg. 2023;52(1):86. 10.1186/s40463-023-00679-w PMC10740346 38135871

[6] Salzano G et al. Pleomorphic adenoma: extracapsular dissection vs. superficial parotidectomy-an updated systematic review and meta-analysis. Med Sci (Basel). 2025;13(3):104. 10.3390/medsci13030104 PMC12372145 40843726

[7] Liu CC, et al. Sensitivity, specificity, and posttest probability of parotid fine-needle aspiration: a systematic review and meta-analysis. Otolaryngol Head Neck Surg. 2016;154(1):9–23. 10.1177/0194599815607841 PMC4896151 26428476

[8] Chen J, et al. Performance of diffusion-weighted imaging for the diagnosis of parotid gland malignancies: a meta-analysis. Eur J Radiol. 2021;134:109444. 10.1016/j.ejrad.2020.109444 33310422

[9] Schmidt RL, et al. A systematic review and meta-analysis of the diagnostic accuracy of fine-needle aspiration cytology for parotid gland lesions. Am J Clin Pathol. 2011;136(1):45–59. 10.1309/AJCPOIE0CZNAT6SQ 21685031

[10] Abend A, et al. Parotid pleomorphic adenoma and apparent diffusion coefficient: a novel clinical prediction tool. Laryngoscope. 2025;135(10):3703–11. 10.1002/lary.32276 40386824

[11] Chakrabarty N, et al. MRI signatures of parotid tumours impacting management decisions: a retrospective study with radiology and pathology correlation. J Med Imaging Radiat Oncol. 2025;69(4):452–61. 10.1111/1754-9485.13865 PMC12175207 40386925

[12] Friedman E, Patino MO, Abdel Razek AAK. MR imaging of salivary gland tumors. Magn Reson Imaging Clin N Am. 2022;30(1):135–49. 10.1016/j.mric.2021.07.006 34802576

[13] Faquin WC, Baloch RE, Barkan Z, Foschini GA, Kurtycz MP, Pusztaszeri DF, Vielh MP. P, The Milan system for reporting salivary gland cytopathology second edition. 2nd ed. Springer; 2023.

[14] Paris J, et al. Preoperative diagnostic values of fine-needle cytology and MRI in parotid gland tumors. Eur Arch Otorhinolaryngol. 2005;262(1):27–31. 10.1007/s00405-003-0730-8 14727124

[15] Geiger JL, et al. Management of salivary gland malignancy: ASCO Guideline. J Clin Oncol. 2021;39(17):1909–41. 10.1200/JCO.21.00449 33900808

[16] Inohara H, et al. The role of fine-needle aspiration cytology and magnetic resonance imaging in the management of parotid mass lesions. Acta Otolaryngol. 2008;128(10):1152–8. 10.1080/00016480701827533 18607904

[17] Bartels S, et al. The relative value of fine-needle aspiration and imaging in the preoperative evaluation of parotid masses. Head Neck. 2000;22(8):781–6. 10.1002/1097-0347(200012)22:8<781::aid-hed6>3.0.co;2-b 11084638

[18] Zaghi S, et al. MRI criteria for the diagnosis of pleomorphic adenoma: a validation study. Am J Otolaryngol. 2014;35(6):713–8. 10.1016/j.amjoto.2014.07.013 25128908

[19] Atay G et al. The role of fine needle aspiration cytology and magnetic resonance imaging in the management of parotid masses. KBB-Forum. 2013;12.

[20] Galli A, et al. Fine needle aspiration cytology for parotid neoplasms: risk of malignancy through inconclusive results and lower grade tumors. Eur Arch Otorhinolaryngol. 2020;277(3):841–51. 10.1007/s00405-019-05733-w 31745630

[21] Suzuki M, et al. Values of fine-needle aspiration cytology of parotid gland tumors: a review of 996 cases at a single institution. Head Neck. 2019;41(2):358–65. 10.1002/hed.25503 30548147

[22] Rossi ED, et al. Second edition of the milan system for reporting salivary gland cytopathology: refining the role of salivary gland FNA. J Am Soc Cytopathol. 2024;13(1):67–77. 10.1016/j.jasc.2023.08.004 38184365

[23] Tryggvason G, et al. Accuracy of fine-needle aspiration and imaging in the preoperative workup of salivary gland mass lesions treated surgically. Laryngoscope. 2013;123(1):158–63. 10.1002/lary.23613 22991236

[24] Yerli H, et al. Diagnosing common parotid tumours with magnetic resonance imaging including diffusion-weighted imaging vs fine-needle aspiration cytology: a comparative study. Dentomaxillofac Radiol. 2010;39(6):349–55. 10.1259/dmfr/15047967 PMC3520240 20729184

[25] Laccourreye O, et al. Positive predictive values of MRI and ultrasound-guided fine-needle aspiration biopsy for isolated parotid swelling in adults: A STARD comparative analysis. Eur Ann Otorhinolaryngol Head Neck Dis. 2022;139(5):269–74. 10.1016/j.anorl.2022.04.004 35538001

[26] Lin W, et al. Differentiation of parotid pleomorphic adenoma from Warthin tumor using signal intensity ratios on fat-suppressed T2-weighted magnetic resonance imaging. Oral Surg Oral Med Oral Pathol Oral Radiol. 2024;137(3):310–9. 10.1016/j.oooo.2023.12.786 38195353

[27] Ikeda K, et al. The usefulness of MR in establishing the diagnosis of parotid pleomorphic adenoma. AJNR Am J Neuroradiol. 1996;17:555–9. PMC8337994 8881252

[28] Fikova A, et al. Utility of fine-needle aspiration biopsy (FNAB) in parotid pleomorphic adenoma diagnosis and management. Biomed Pap Med Fac Univ Palacky Olomouc Czech Repub. 2024;168(2):156–61. 10.5507/bp.2023.027 37431621

[29] Shkedy Y, et al. Fine-needle aspiration cytology for parotid lesions, can we avoid surgery? Clin Otolaryngol. 2018;43(2):632–7. 10.1111/coa.13038 29178364

[30] Heaton CM, et al. Pleomorphic adenoma of the major salivary glands: diagnostic utility of FNAB and MRI. Laryngoscope. 2013;123(12):3056–60. 10.1002/lary.24247 23798340

[31] Maraghelli D, et al. Magnetic resonance imaging of salivary gland tumours: key findings for imaging characterisation. Eur J Radiol. 2021;139:109716. 10.1016/j.ejrad.2021.109716 33866123

[32] Habermann CR, et al. Diffusion-weighted echo-planar MR imaging of primary parotid gland tumors: is a prediction of different histologic subtypes possible? Am J Neuroradiol. 2009;30(3):591. 10.3174/ajnr.A1412 PMC7051445 19131405

[33] Munhoz L, et al. Application of diffusion-weighted magnetic resonance imaging in the diagnosis of salivary gland diseases: a systematic review. Oral Surg Oral Med Oral Pathol Oral Radiol. 2019;128(3):280–310. 10.1016/j.oooo.2019.02.020 31029591

[34] Celebi I, et al. Quantitative diffusion-weighted magnetic resonance imaging in the evaluation of parotid gland masses: a study with histopathological correlation. Clin Imaging. 2013;37(2):232–8. 10.1016/j.clinimag.2012.04.025 23465973

[35] Kato H, et al. Salivary gland tumors of the parotid gland: CT and MR imaging findings with emphasis on intratumoral cystic components. Neuroradiology: J Dedic Neuroimaging Interventional Neuroradiol. 2014;56(9):789–95. 10.1007/s00234-014-1386-3 24948426

[36] Coudert H, et al. Multiparametric magnetic resonance imaging of parotid tumors: a systematic review. Diagn Interv Imaging. 2021;102(3):121–30. 10.1016/j.diii.2020.08.002 32943368

[37] Moons KG, et al. Transparent Reporting of a multivariable prediction model for Individual Prognosis or Diagnosis (TRIPOD): explanation and elaboration. Ann Intern Med. 2015;162(1):W1–73. 10.7326/M14-0698 25560730

